# *In vitro* biological evaluation and *in silico* insights into the antiviral activity of standardized olive leaves extract against SARS-CoV-2

**DOI:** 10.1371/journal.pone.0301086

**Published:** 2024-04-25

**Authors:** Taghreed A. Majrashi, Mahmoud A. El Hassab, Sara H. Mahmoud, Ahmed Mostafa, Engy A. Wahsh, Eslam B. Elkaeed, Fatma E. Hassan, Wagdy M. Eldehna, Shimaa M. Abdelgawad

**Affiliations:** 1 Department of Pharmacognosy, College of Pharmacy, King Khalid University, Asir, Saudi Arabia; 2 Department of Medicinal Chemistry, Faculty of Pharmacy, King Salman International University (KSIU), South Sinai, Egypt; 3 Center of Scientific Excellence for Influenza Viruses, National Research Centre, Giza, Egypt; 4 Clinical Pharmacy Department, Faculty of Pharmacy, October 6 University, Giza Governorate, Egypt; 5 Department of Pharmaceutical Sciences, College of Pharmacy, AlMaarefa University, Riyadh, Saudi Arabia; 6 Department of Physiology, General Medicine Practice Program, Batterjee Medical College, Jeddah, Saudi Arabia; 7 Medical Physiology Department, Kasr Alainy, Faculty of Medicine, Cairo University, Giza, Egypt; 8 Department of Pharmaceutical Chemistry, Faculty of Pharmacy, Kafrelsheikh University, Kafrelsheikh, Egypt; 9 Pharmacognosy Department, Faculty of Pharmacy, Fayoum University, Fayoum, Egypt; Concordia University, CANADA

## Abstract

There is still a great global need for efficient treatments for the management of SARS-CoV-2 illness notwithstanding the availability and efficacy of COVID-19 vaccinations. Olive leaf is an herbal remedy with a potential antiviral activity that could improve the recovery of COVID-19 patients. In this work, the olive leaves major metabolites were screened *in silico* for their activity against SARS-CoV-2 by molecular docking on several viral targets such as methyl transferase, helicase, Pl^pro^, M^pro^, and RdRp. The results of *in silico* docking study showed that olive leaves phytoconstituents exhibited strong potential antiviral activity against SARS-CoV-2 selected targets. Verbacoside demonstrated a strong inhibition against methyl transferase, helicase, Pl^pro^, M^pro^, and RdRp (docking scores = -17.2, -20, -18.2, -19.8, and -21.7 kcal/mol.) respectively. Oleuropein inhibited 5rmm, M^pro^, and RdRp (docking scores = -15, -16.6 and -18.6 kcal/mol., respectively) respectively. Apigenin-7-*O*-glucoside exhibited activity against methyl transferase and RdRp (docking score = -16.1 and -19.4 kcal/mol., respectively) while Luteolin-7-*O*-glucoside inhibited Pl^pro^ and RdRp (docking score = -15.2 and -20 kcal/mol., respectively). The *in vitro* antiviral assay was carried out on standardized olive leaf extract (SOLE) containing 20% oleuropein and IC_50_ was calculated. The results revealed that 20% SOLE demonstrated a moderate antiviral activity against SARS-CoV-2 with IC_50_ of 118.3 *μ*g /mL. Accordingly, olive leaf could be a potential herbal therapy against SARS-CoV-2 but more *in vivo* and clinical investigations are recommended.

## 1. Introduction

Throughout the history, humans were infected by several viruses and, up to date, billions of peoples around the world have died because of different viral infections [1]. The current COVID-19 pandemic, which is being driven by the SARS-CoV-2 new coronavirus, has significantly increased morbidity and death globally and has had a significant impact on all facets of human existence [1, 2]. An infection of the lower respiratory tract known as COVID-19 can cause pneumonia after starting off with flu-like symptoms such as sore throat, fever, headache, lethargy, and malaise. Additionally, back pain, diarrhea, and a loss of taste and smell were noted [3].

As of 19 July 2023, the WHO estimates that there have been 768,237,788 COVID-19 cases globally, with 6,951,677 deaths (https://covid19.who.int/). Regarding the effectiveness and safety of the vaccines that are currently on the market, little information is available. Up to date, the FDA approved only one drug (Paxlovid^®^), for the management of COVID-19 infection [3]. Therefore, the development of various antiviral medications is essential for controlling the infection. WHO reported that around 80% of the populations throughout the developing countries have used medicinal plants to treat their illnesses [4, 5]. Traditionally, natural products were used to treat many viral diseases [4, 6].

Oleuropein, the main chemical in the olive leaves extract, has been shown to have antiviral properties against the viral hemorrhagic septicemia virus (VHSV) [7], herpes mononucleosis, hepatitis virus, rotavirus, bovine rhinovirus, canine parvovirus, feline leukemia virus [8], and HIV-1 [7, 9, 10]. In addition, respiratory viruses, including the syncytial virus and type 3 para-influenza virus were significantly inhibited by olive leaves [11].

Regarding COVID-19 infection, several studies discussed the potential value of olive leaf extract or its phytochemicals in the management of COVID-19 infection. Several studies reported the *in silico* antiviral activities of oleuropein and other phyto-constituents in olive leaves such as hydroxytyrosol, oleanolic acid, maslinic acid, luteolin, luteolin-7-*O*-glucoside, verbascoside, apigenin-7-*O*-glucoside and kaempferol against several targets such as SARS-CoV-2 viral proteases (Mpro/3CLpro, PLpro), TLRs, ACE2, RBD, NSP15, HSPA5 SBD*β*, TMPRSS2, S protein and Furin [3, 12–18]. Some of the olive leaves constituents such as hydroxytyrosol, luteolin, and kaempferol showed antiviral properties against SARS-CoV-2 *in vitro* [3, 18–21].

Despite of the available data on the activity of olive compounds against SARS-CoV-2, no studies evaluated the real antiviral effect of the olive leaf extract in the inhibition of SARS-CoV-2 and controlling the infection. Therefore, the objective of this study is to assess the antiviral efficacy of commercially available, standardized olive leaf capsules using *in silico* and *in vitro* screening assays in order to determine whether they are useful as a natural supplement for management of COVID-19 infection.

Several key targets in the life cycle of the virus have been suggested as potential candidates for the development of targeted antiviral drugs (DAAs) against COVID-19. These include the non-structural protein 12 (nsp12), an RNA-dependent RNA polymerase (RdRp) crucial for replicating the virus genome. Additionally, the 3C-like protease (3CLpro) and papain-like protease (PLPro) are essential targets, playing a critical role in the SARS-CoV-2 replication cycle by processing the polyprotein produced during transcription into functional subunits. To this end, we have screened the major compounds in the extract against the previously mentioned targets [22–24].

## 2. Material and methods

### 2.1. *In silico* study

All docking studies in this work were carried out using the Molecular operating environment (MOE 2019.02) software [25, 26]. SARS-CoV-2 main targets: methyl transferase, helicase, papin-like protease (Pl^pro^), main protease (M^pro^), and RNA-dependent-RNA polymerase (RdRp) X-ray crystal structures were retrieved from the protein data bank using the PDB IDs 6YZ1, 5RMM, 7JRN, 7L8J, AND 7ED5, respectively. The hydrogens and charges of the receptors were initially adjusted using AMBER10: EHT incorporated in MOE software. Each of the five targets’ active site was determined by building 4.5 Å around the bound co-crystalized ligands in the active site. MOE2019’s 2D builder was used to sketch the five major compounds of the olive, which were then converted to 3D structures using the same program. Finally, MOE developed 2D and 3D interaction diagrams to examine the docking outcomes.

### 2.2. *In vitro* study

#### 2.2.1. Preparation of plant material

A standardized 750 mg olive leaf capsule containing 20% Oleuropein (150 mg Oleuropein/ capsule) were purchased from Health Harmony (https://www.amazon.com/Olive-Extract-NON-GMO-Super-Strength/dp/B01KVADU1O?ref_=ast_sto_dp). Two capsules were extracted with 80% methanol (50 mL×3) using a rotary evaporator (BUCH, rota vapor^®^ R-300) and the extract was concentrated after being filtered out. Residue was weighed and stored in a refrigerator at -4°C until used.

#### 2.2.2. Cytotoxicity assay

Stock solutions of the test chemicals in 10% DMSO in ddH_2_O were generated in order to determine the half maximum cytotoxic concentration (CC_50_), and these solutions were then diluted further to the working solutions with DMEM. The 3-(4, 5-dimethylthiazol-2-yl)-2, 5-diphenyltetrazolium bromide (MTT) technique was used to examine the extracts’ cytotoxic activity in VERO-E6 cells. Briefly, the cells were seeded and incubated for 24 h at 37 °C in 5% CO_2_ in 96 well-plates (100 *μ*l/well at a density of 3×10^5^ cells/ml). Cells were given treatment with varying doses of the investigated substances in triplicates after 24 hours. After discarding the supernatant after 24 hours, the cell monolayers were washed three times with sterile 1x PBS before being treated with MTT solution (20 *μ*l of a 5 mg/ml stock solution) and incubated at 37 °C for 4 hours before medium aspiration. The produced formazan crystals were dissolved in 200 μl of acidified isopropanol (0.04 M HCl in absolute isopropanol = 0.073 ml HCL in 50 ml isopropanol) in each well. A multi-well plate reader was used to measure the absorbance of formazan solutions at *λ* max of 540 nm with 620 nm serving as a reference wavelength. The percentage of cytotoxicity compared to the untreated cells was determined with the following equation [27].

The concentration that displayed 50% cytotoxicity was determined using a plot of percent cytotoxicity against sample concentration (CC_50_).


$$\%\;cytotoxicity=\frac{\left(absorbance\;of\;cells\;without\;treatment-absorbance\;of\;cells\;with\;treatment\right)\;X\;100}{absorbance\;of\;cells\;without\;treatment\;}$$

### 2.3. Inhibitory concentration 50 (IC_50_) determination

Vero-E6 cells (2.4×10^4^) were evenly spread among 96-well tissue culture plates before being incubated overnight at 37°C with 5 percent CO_2_. Following a single PBS wash, the cell monolayers were exposed to hCoV-19/Egypt/NRC-03/2020 (GSAID accession number: EPI ISL 430820) for one hour at room temperature (RT). Additional 100 *μ*l of DMEM with different doses of the test substances was applied on top of the cell monolayers. The cells were fixed with 100 *μ*l of 4 percent paraformaldehyde for 20 minutes and stained with 0.1 percent crystal violet in distilled water for 15 minutes at room temperature following 72 hours of incubation at 37°C in an incubator with 5% CO_2_. The optical density of the color was then measured at 570 nm using an 200rt plate reader (Anthos Labtec Instruments, Heerhugowaard, Netherlands) after the crystal violet dye had been dissolved using 100 *μ*l of absolute methanol each well. The amount of the chemical called the inhibitory concentration (IC_50_) needed to diminish the virus-induced cytopathic effect (CPE) by 50% in comparison to virus control [27].

### 2.4. Statistical analyses

All experiments were performed in triplicates. GraphPad Prism 5.01 software was used to perform statistical tests and graphical data presentation. Data are presented as the mean’s average. The values of IC_50_ and CC_50_ curves which were determined using GraphPad prism as the "best fit value," represent the nonlinear fit of "normalise" and "transform" of the provided data.

## 3. Results

### 3.1. Results of *in silico* molecular docking study

*In silico* molecular docking studies have played a very important role in discovery of biological activities of natural metabolites [28–32].

Reviewing the previous literature; the major phyto-constituents in olive leaves was reported such as oleuropein, hydroxytyrosol, verbascoside, luteolin, luteolin-7-*O*-glucoside, and apigenin-7-*O*-glucoside [33]. Olive leaves extract is usually standardized to its oleuropein content usually up to 20% (w/w), flavonoids which constitutes up 1.8% (w/w), of which 0.8% is luteolin 7-glucoside [34], while Apigenin-7-O-glucoside was found in concentration up to 2.05 mg/g olive leaf extract [35]. Hydroxytyrosol in addition was previously determined in olive leaf extracts in concentration up to 2.28 mg/100 g leaf extract [36], and verbascoside content up to 3.97 mg/g olive leaf extract [35].

The purpose of this section is to evaluate the antiviral potential of major phyto-constituents in olive leaves such as oleuropein, hydroxytyrosol, verbascoside, luteolin, luteolin-7-*O*-glucoside, and apigenin-7-*O*-glucoside, using *in silico* molecular docking technique. Accordingly, five key enzymes in the SARS-CoV-2 life cycle were identified as prospective targets for olive extract, namely methyl transferase, helicase, Pl^pro^, M^pro^, and RdRp. The selected targets’ 3D structural coordinates were retrieved from the protein data bank using the following IDs: 6YZ1, 5RMM, 7JRN, 7L8J, AND 7ED5 for methyl transferase, helicase, Pl^pro^, M^pro^, and RdRp, respectively. Interestingly, the five compounds achieved excellent docking scores with all the five targets (Table 1).

**Table 1:** Docking scores (kcal/mol.) of olive leaves compounds against selected targets of SARS-CoV-2.

Compound name	Methyl transferase (6YZ1)	Helicase (5RMM)	Pl^pro^ (7JRN)	M^pro^ (7L8J)	RdRp (7ED5)
Oleuropein	-14	-15	-14.8	-16.6	-18.6
Hydroxytyrosol	-8.2	-9.7	-9.7	-10.5	-12.4
Verbascoside	-17.2	-20	-18.2	-19.8	-21.7
Apigenin-7-*O*-glucoside	-16.1	-13.6	-13.2	-12.9	-19.4
Luteolin-7-*O*-glucoside	-13.1	-13.7	-15.2	-12.8	-20
Co-crystalized ligand	-16.8				
Co-crystalized ligand		-21.2			
Co-crystalized ligand			-17.9		
Co-crystalized ligand				-22.3	
Co-crystalized ligand					-22.7

**Fig 1:**
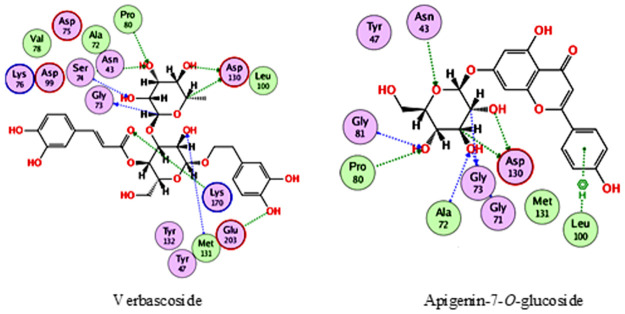
2D interactions of verbascoside and apigenin-7-*O*-glucoside with methyl transferase.

Inspecting the docking results with methyl transferase, revealed that verbascoside and apigenin-7-*O*-glucoside were the best compounds, achieving docking scores of -17.2 and -16.1 kcal/mol., respectively. As Fig 1 that reveals the interaction of verbascoside and apigenin-7-*O*-glucoside with methyl transferase, verbascoside was able to interact with Asn43, Gly73, Ser74, Pro80, Asp130, Met131, Lys170 and Glu203, while apigenin-7-*O*-glucoside interacted with Asn43, Gly71, Ala72, Gly73, Pro80, Gly81, Leu100 and Asp130.

**Fig 2:**
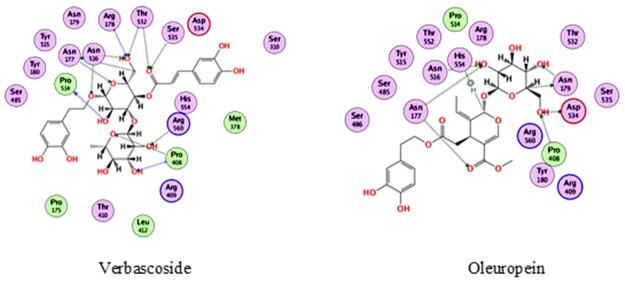
2D interactions of verbascoside and oleuropein with helicase.

In the docking with helicase, verbascoside and oleuropein were the best compounds, achieving docking scores of -20 and -15 kcal/mol., respectively. As depicted from Fig 2, verbascoside was able to bind to helicase through interacting with Asn177, Arg178, Pro408, Pro514, Asn516, Thr532, Ser535 and Arg560, while oleuropein interacted with Asn177, Asn179, Pro408, Asn516, Asp534, and His554.

**Fig 3:**
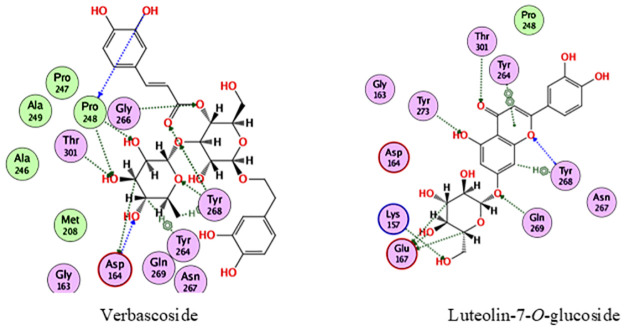
2D interactions of verbascoside and luteolin-7-O-glucoside with Pl^pro^.

In the docking with Pl^pro^, verbascoside and luteolin-7-*O*-glucoside were the best compounds, achieving docking scores of -18.2 and -15.2 kcal/mol., respectively. As depicted from Fig 3, verbascoside was able to bind to Pl^pro^ through interacting with Asp164, Pro248, Tyr264, Gly266, Tyr268 and Thr301, while luteolin-7-*O*-glucoside interacted with Lys157, Glu167, Tyr264, Gln269, Tyr268, Tyr273 and Thr301.

**Fig 4:**
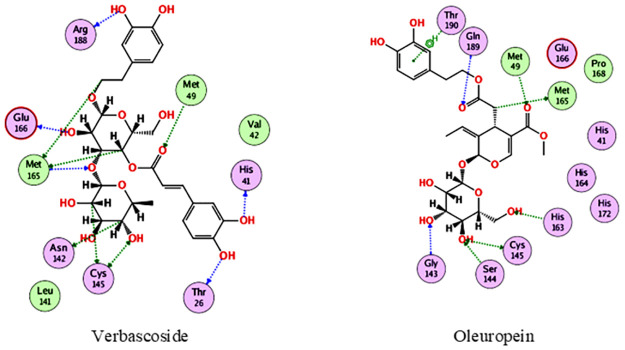
2D interactions of verbascoside and oleuropein with M^pro^.

In the docking with M^pro^, verbascoside and oleuropein were the best compounds, achieving docking scores of -19.8 and -16.6 kcal/mol., respectively. As depicted from Fig 4, verbascoside was able to bind to M^pro^ through interacting with Thr26, His41, Met49, Asn142, Cys145, Met165, Glu166 and Arg188, while oleuropein interacted with Met49, Gly143, Ser144, Cys145, His163, Met165, Gln189 and Tyr190.

**Fig 5:**
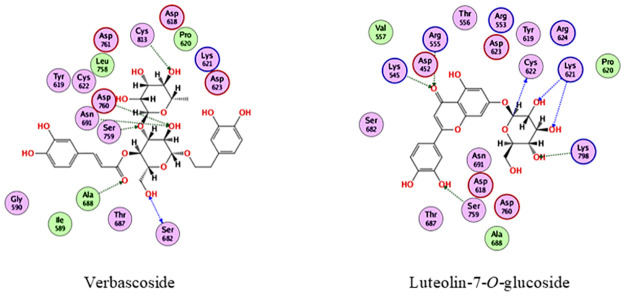
2D interactions of verbascoside and luteolin-7-*O*-glucoside with RdRp.

In the docking with RdRp, verbascoside and luteolin-7-*O*-glucoside were the best compounds, achieving docking scores of -21.7 and -20 kcal/mol., respectively. As depicted from Fig 5, verbascoside was able to bind to RdRp through interacting with Ser682, Ala688, Asn691, Ser759, Asp760, and Cys812, while luteolin-7-*O*-glucoside interacted with Lys545, Arg555, Lys621, Cys622, Ser759 and Lys798. To this end, it is suggested that the olive extract components have synergistic effect in inhibiting SARS CoV-2.

### 3.2. Results of *in vitro* antiviral assay

**Fig 6:**
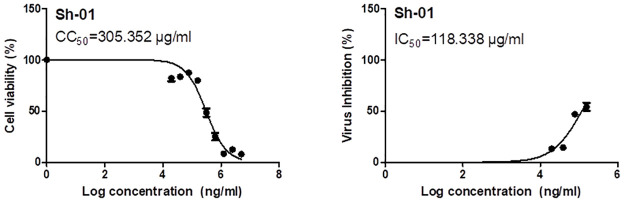
The dose-response and inhibition curves for the 20% standardized olive leaf extract, which were determined using GraphPad Prism’s nonlinear regression analysis, show the half-maximal cytotoxic concentration (CC_50_) in Vero E6 cells and the inhibitory concentration 50 percent (IC_50_) against NRC-03-nhCoV.

The half-maximal cytotoxic concentration, or "CC_50_," was calculated using a crystal violet assay to determine the proper concentrations to define the antiviral activity of the 20% SOLE (Fig 6). The extract demonstrated a wide range of safety within the tested concentrations (10 ng/mL-100 mg/mL). The 20% SOLE showed a weak inhibitory action against NRC-03-nhCoV, with an IC_50_ of 118.3 *μ*g /mL, according to the antiviral screening.

## 4. Discussion

As a part of our continuous effort of finding natural products-based anti-SARS-CoV-2 therapeutics, we investigated the antiviral potential of major phytoconstituents in olive leaves such as oleuropein, hydroxytyrosol, verbascoside, luteolin, luteolin-7-*O*-glucoside, and apigenin-7-*O*-glucoside, using *in silico* molecular docking against methyl transferase, helicase, Pl^pro^, M^pro^, and RdRp.

Our rationale in the present research was based on the hypothesis established by Abdelgawad et al [3] for the potential benefit of olive leaf extract for COVID-19 patients where the *in silico*, *in vitro* and *in vivo* antiviral studies (anti SARS-CoV-2), about olive compounds were summarized and discussed. The antiviral activity of olive leaf metabolites against SARS-CoV-2 were reported in several *in silico* studies against several viral targets such as Mpro/3CLpro, PLpro, TLRs, ACE2, RBD, NSP15, HSPA5 SBD*β*, TMPRSS2, S protein and Furin [3]. In this study, other targets are screened such as methyl transferase, helicase and RdRp.

Oleuropein has been tested in a previous study by Erman Salih İSTİFLİ1 [37] against SARS-CoV-2 M^pro^ and RdRp (docking scores = -7.0 and -8.0 kcal mol^-1^, respectively). However, our findings demonstrated higher affinity of oleuropein toward these targets as represented in Table 1.

Verbascoside showed previously high docking scores (-14.4, − 9.1317, and −9.361) toward Pl^pro^, M^pro^, and RdRp respectively [17, 38]. While our results showed higher affinity of verbascoside to these target as represented in Table 1.

Apigenin-7-*O*-glucoside [36] was previously reported for its *in silico* potential inhibition of M^pro^ (with a binding energy of −8.2 kcal/mol.) that is lower than our finding as in Table 1.

Several computational studies discussed the antiviral activities of olive leaves phytoconstituents against SARS-CoV-2 [3, 12–18, 39–41]. However, to our knowledge, no study reported the *in vitro* antiviral effect of olive leaf extract against SARS-CoV-2. So, our findings represent the first *in vitro* antiviral effect of SOLE against SARS-CoV-2.

Despite the strong effect of the tested compounds throughout the *in silico* screening, the *in vitro* assay demonstrated weak antiviral effect. The *in silico* effect of compounds can rationalize the *in vitro* activity. However, the weak *in vitro* antiviral effect may be explained by antagonism effect when all active compounds present together in the extract or due to presence of other compounds in the extract, but this still need further investigations.

As discussed in the present study, phytoconstituents from olive leaves, could be considered as promising anti-SARS-CoV-2 leads that can suppress its activity, and olive leaves could be used as a supplement in COVID-19 patients for their antiviral effect demonstrated in this study alongside to its reported *in vivo* anti-inflammatory and immunomodulatory activities [3].

## 5. Conclusion

In this study, we have highlighted the importance of olive leaves phytoconstituents as a promising candidate metabolite for developing SARS-CoV-2 methyl transferase, helicase, proteases, and RNA-dependent-RNA polymerase inhibitors. Promisingly, verbacoside, oleuropein, apigenin-7-*O*-glucoside and luteolin-7-*O*-glucoside significantly inhibited the tested targets. Additionally, SOLE (20% oleuropein) demonstrated a weak antiviral activity against SARS-CoV-2 with IC_50_ of 118.3 *μ*g /mL. In conclusion, olive leaf could be a potential herbal supplement against SARS-CoV-2 but more *in vivo* and clinical investigations are recommended.
